# Design and validation of a questionnaire to assess organizational culture in French hospital wards

**DOI:** 10.1186/s12913-016-1736-4

**Published:** 2016-09-17

**Authors:** F. Saillour-Glénisson, S. Domecq, M. Kret, M. Sibe, J. P. Dumond, P. Michel, the TheOReM group

**Affiliations:** 1 CHU de Bordeaux – Institut de Santé Publique d’Epidémiologie et de Développement, 146 rue Léo Saignat, 33076 Bordeaux Cedex, France; 2 Comité de Coordination de l’Evaluation Clinique et de la Qualité en Aquitaine, Hôpital Xavier Arnozan, 33604 Pessac Cedex, France; 3 Institut de Santé Publique et de Développement, 146 rue Léo Saignat, 33076 Bordeaux Cedex, France; 4 Université Paris-Est Créteil Val de Marne (UPEC), Faculté de sciences économiques et de gestion, Place de la Porte des Champs, 4 Route de Choisy, 94010 Créteil Cedex, France; 5 Hospices Civils de Lyon, Université Claude Bernard Lyon 1, 3, quai des Célestins, 69002 Lyon, France

**Keywords:** Organisational culture, Hospital, Management, Validation, Questionnaire

## Abstract

**Background:**

Although many organizational culture questionnaires have been developed, there is a lack of any validated multidimensional questionnaire assessing organizational culture at hospital ward level and adapted to health care context. Facing the lack of an appropriate tool, a multidisciplinary team designed and validated a dimensional organizational culture questionnaire for healthcare settings to be administered at ward level.

**Methods:**

A database of organizational culture items and themes was created after extensive literature review. Items were regrouped into dimensions and subdimensions (classification validated by experts). Pre-test and face validation was conducted with 15 health care professionals.

In a stratified cluster random sample of hospitals, the psychometric validation was conducted in three phases on a sample of 859 healthcare professionals from 36 multidisciplinary medicine services: 1) the exploratory phase included a description of responses’ saturation levels, factor and correlations analyses and an internal consistency analysis (Cronbach’s alpha coefficient); 2) confirmatory phase used the Structural Equation Modeling (SEM); 3) reproducibility was studied by a test-retest.

**Results:**

The overall response rate was 80 %; the completion average was 97 %. The metrological results were: a global Cronbach’s alpha coefficient of 0.93, higher than 0.70 for 12 sub-dimensions; all Dillon-Goldstein’s rho coefficients higher than 0.70; an excellent quality of external model with a Goodness of Fitness (GoF) criterion of 0.99. Seventy percent of the items had a reproducibility ranging from moderate (Intra-Class Coefficient between 50 and 70 % for 25 items) to good (ICC higher than 70 % for 33 items).

**Conclusions:**

COMEt (Contexte Organisationnel et Managérial en Etablissement de Santé) questionnaire is a validated multidimensional organizational culture questionnaire made of 6 dimensions, 21 sub-dimensions and 83 items. It is the first dimensional organizational culture questionnaire, specific to healthcare context, for a unit level assessment showing robust psychometric properties (validity and reliability). This tool is suited for research purposes, especially for assessing organizational context in research analysing the effectiveness of hospital quality improvement strategies. Our tool is also suited for an overall assessment of ward culture and could be a powerful trigger to improve management and clinical performance. Its psychometric properties in other health systems need to be tested.

**Electronic supplementary material:**

The online version of this article (doi:10.1186/s12913-016-1736-4) contains supplementary material, which is available to authorized users.

## Background

The concept of Organizational Culture (OC) has become a major element in organizational research, because it is one determinant of individual behaviour and can be linked with some organizational outcomes as job satisfaction, professional engagement, turn over or achievement of goals [1–6]. In addition, operational studies tend to confirm the links between culture and performance in the health care sector, as for example quality of care, promptness of care, external stakeholders satisfaction, patient satisfaction and mortality rate [7–10]. Moreover, two landmark reports from the Institute of Medicine and calls to reforms of the National Health Service (NHS) have stressed the need for cultural changes in order to deliver improvements in quality and performance [11–14]. It is believed that only a transformation of professional and organizational culture will enable the instillation of new values, beliefs and assumptions to guide and underpin new way of working in healthcare organizations, for examples developing coordination of care across patient-conditions and services, improving cooperation among clinicians, deploying knowledge and information sharing, evidence-based clinical decisions making and the use of information technology.

However, the literature on the link between OC and quality of care is controversial. In their literature review on the link between OC and performance, T Scott et al. [8] identified four studies finding plausible evidence for a link between culture and performance, four with little evidence for such a link and two providing unclear findings. If many contextual social, economic, political and methodological reasons can explain some of these discrepancies [15], some authors [16–18] suggest that one possible explanation for the difficulty in finding consistent relationship between culture and effectiveness is that culture may influence effectiveness indirectly. They propose that culture potentially has a direct effect on attitudinal factors such as morale, commitment, job satisfaction, and that these “intermediate” factors then directly impact effectiveness. So, as the relationships between culture and performance are multiple, complex and contingent, we actually need tool allowing the global comprehension of the local organizational context making it possible to understand not only the “if” but also the “how” managerial practices could influence performance in health care settings [4–9]. Consistently, B Schneider et al. call for tools that are able to assess the moderator value of OC on the relationship between OC and performance [15].

Two main schools of thought can be distinguished, to divide the numerous definitions, meaning and the constitutive elements of OC [19–22]. A first approach considers culture as something that an organization “is”. Here, culture serves as a metaphor for describing an organization rather than being seen as something readily identifiable or separate from the organization itself. In contrast, the second perspective considers culture as something that an organization “has”. That is, culture represents aspects or variables of the organization that can be isolated, described, and manipulated. Under this latter conception, there is agreement about the OC definition, as shared values, common understandings, common beliefs and expectations of people belonging to a working team [23–25], that underpin and reinforce their behaviours and distinguish them from other teams [26]. OC encapsulates not only what members of the organization have learned but also what they believe. It comprises perceptions as well as practices shared within the organization, rather than being solely based on values held by individual members [27]. Schein identifies three layers for OC: at the most basic layer are the underlying assumptions that are the basic “taken for granted” beliefs that structure the thinking and behaviour of an individual; the second level, or values, constitutes the basic foundations for making judgements and distinguishing “right” from “wrong” behaviour; the third level corresponds to artefacts including the physical and behavioural manifestations of culture.

There is also variation in the types of assessment approaches used to analyse OC (qualitative versus quantitative). A commonly used method is the survey. There are many different types of questionnaires differing in terms of whether they are either typological, i.e. based on predefined cultural types with assessment results in one or more “types” of OC (for examples: clan, adhocracy, hierarchy or market cultures in the Competing Value Framework [28]) or dimensional, i.e. based on a dimensional patterns (for example: leadership, social life and communication, management…) which describes a culture by its position on a number of conditions variables [29]. Typological questionnaires allowing a global view of organizational culture have been often validated at institutional (hospital) level. They constitute summative instruments defining the dominant culture of a setting. On the other hand, dimensional instruments allow precise and detailed description of the different OC components in a setting. They constitute effective operational diagnostic tools for a realistic analysis of managerial practices, which can be useful for guiding an intervention [4]. They are also good research instruments assessing the attitudinal factors needed for understanding the mechanism of OC effectiveness on quality of care. The OC questionnaires also vary in scope, some focusing on the assessment in one or more specific domains of organizational culture [30–32] and others assessing a more comprehensive range of issues [8]. They also vary according to the level of culture they tap into, with none convincingly addressing the deeper underlying assumptions that guide attitudes and behaviour and inform the stable substrate of culture [8].

An instrument that works well for one investigation may not be so effective for another study. In this regard, the concept of “fitness for purpose” has gained increasing importance within the health measurement literature and requires the evaluation of the quality of a given assessment instruments to be conducted in the context of its application, audience and intended use [33, 34].

CCECQA (Comité de Coordination de l’Evaluation Clinique et de la Qualité en Aquitaine), in partnership with French research teams in management, initiated and coordinated a national project, the TheOReM© project (Organizational Theories, Recommendations and Management) funded by the HAS (Haute Autorité de Santé - French National Authority for Health). The aim of the study was to identify the managerial and organisational characteristics of medical wards that are most strongly associated with performance. A part of the managerial and organisational characteristics were assessed by an OC questionnaire. The analysis level was at the level of the ward or unit, i.e. the smallest management entity at hospital, constituting teams of professionals sharing daily patient management practices. The ward has been shown to be an effective level for OC assessment and for quality and security improvement [35, 36].

For our purpose, a detailed and comprehensive managerial and organizational diagnostic tool, validated at ward level, was needed. OC typological questionnaires were excluded, as not descriptive enough for our needs. Existing OC dimensional organizational questionnaires did not show robust psychometric performance, or, adapted from industrial contexts, were not considered as specific enough for our need [37–39]. T Jung et al’s review of the psychometric properties of 48 OC tools noted that less than half (46 %) of the instruments had published data demonstrating adequate internal consistencies. Additionally, only one in five (21 %) instruments demonstrated adequate evidence for aggregating individual data to be representative of the organizational as a whole. Lastly, T Jung et al’s review noted that only one in five (19 %) of the examined instruments presented adequate evidence of the dimensionality of the instrument. Moreover, the existing OC questionnaires suffer from a lack of construction relying on a generally accepted and common framework that allows for a consistent approach to the conceptualization and measurement of organizational culture.

For all these reasons, the TheOReM© working group decided to design and validate a dimensional questionnaire relevant at healthcare ward level in public or private settings, on the basis of predefined dimensional patterns.

This paper presents the development and validation of this tool called COMEt (Contexte Organisationnel et Managérial en Etablissement de santé).

## Methods

### Design of a questionnaire

#### Selection of measuring instruments

A comprehensive literature search was conducted to identify existing measuring instruments. The retrieval strategy (data bases explored and key terms used) is shown in Table 1. References indicated in articles found were explored and experts were contacted.

**Table 1:** Data bases explored and key word used for the comprehensive literature search for existing OC measuring instruments

Databases explored	Key words
Sciences Direct, Medline, Eric-Francis, Pascal, The National Institute Of Research And Security Data Base, Business Source Premier	First step “Organizational Culture” Or “Organizational Climate” Or “Organizational Commitment” Or “Job Satisfaction” Or “Leadership” Or “Psychological Contract” Or “Decision Style” Or “Trust” Or “Organizational Citizenship Behaviour” Or “Employee-Organisation Relationship”;
	Second step “Organizational Culture” Or “Professional Culture” And “Hospital”

Papers inclusion criteria were: French or English language, date of publication between 1980 and 2014, description of a quantitative organizational culture measurement instrument, based on a typology or not, available in its entirety or not, free or on a paying basis. Out of this first selection, a multidisciplinary expert group, composed of four methodologists and three specialists in management sciences, working in three different French research departments (Bordeaux, Paris, Nantes) specialised in questionnaire validation or management sciences, selected tools on the following criteria: quantitative instruments of organizational culture, having already been used in a healthcare settings, having a good face validity to assess a broad range of cultural dimensions. Priority was given to those instruments for which some data were available on their statistical validity and reliability as measures of organizational culture.

#### Definition of dimensions and sub-dimensions to be explored

A database including all items and themes of selected instruments was created. From that base, the expert group established by consensus the organizational culture dimensions and sub-dimensions to be explored. During three expert meetings, organizational culture dimensions and sub-dimensions were determined on the basis of OC concept and defined; all the database items and themes were classified in each of them.

#### Item formulation

The items identified in the chosen instruments were classified into specified dimensions and sub-dimensions. Redundant or irrelevant items to the French health system were excluded. The wording of items was in French with a special attention to the consistency in the editing. Items should have the same formulation. They all were edited as affirmative sentences addressing directly to the questioned professional or to the community of professionals of the unit. Whenever possible, validated scales were introduced into the questionnaire. The response options were a five point Likert scale, from “strongly disagree” to “strongly agree”.

#### Content validity

Cross validation of items classification in predefined dimensions and sub-dimensions was done by the expert group. In addition, two organisation sociologists reviewed the questionnaire and confirmed the assumption that: all the items were related to the ward’s OC, each item was belonging to one of the dimensions and each dimension was represented by one or several items.

#### Pre-test and face validity

Finally, some 15 field health care professionals, from public and private hospital wards, completed the questionnaire and assessed its applicability and acceptance level.

### Validation of the questionnaire

#### Data collection

A survey was conducted in five French regions: Aquitaine, Bretagne, Franche-Comté, Poitou-Charentes, Rhône-Alpes, representing a population of 10 millions inhabitants.

The study population consisted of all professionals involved in patient care in the participating medical wards (doctors, nurses and orderlies), during the daytime or at night, and whatever their status or length of service. The following were excluded: trainee nurses and orderlies, doctors who were working only temporarily in the department, other paramedical personnel (physiotherapists, psychologists, etc.), and professional staff on extended leave. Each professional received oral and written information about the study, delivered during a meeting organized in each participating ward. They gave all verbal consent to participate.

The group of wards participating in the study was randomly chosen in each region, using a stratified cluster sample design. Four strata were defined, according to type of hospital: 1) university teaching hospitals called university hospital; 2) non-university public hospitals with more than 350 beds called large hospital; 3) non-university public hospitals with fewer than 350 beds called small hospital; and 4) private hospitals. If an hospital declined to participate, a new one was selected from the randomized list until there was a sufficient number of hospitals in each stratum.

Within the included wards, the study population of health care professionals was asked to complete the self administered OC questionnaire. The group of health care professionals having filled in the questionnaire constituted the study sample.

The data collection procedure ensured confidentiality of responses; it was coordinated in each region by one member of the study group.

#### Statistical analyses

The psychometric properties of the questionnaire were assessed in three successive steps: a) exploratory analysis, b) confirmatory analysis and c) reproducibility [40, 41]. The programs used were Stata and XL-Stat [42].

- Exploratory analysis presented first the item response rate, the distribution of the responses for comprehensiveness and saturation assessment (floor and ceiling effects) and symmetry of the distribution. Dimensional structure was studied using the Spearman's correlation matrix and the Principal Component Analysis (PCA). The correlation analysis identified redundant items (correlation higher than 0.80), and wrongly located or irrelevant ones (correlation lower than 0.20). PCA allowed to identify grouping of items, called factors, and to check the one-dimensional character of the questionnaire. The number of significant factors was determined by applying the combination of criteria (the Kaiser’s criterion- eigenvalue > 1- and the Horn parallel analysis) and an Oblimin rotation was used to facilitate the factor interpretation. Each factor was described taking into account a squared cosine higher than 0.30 indicating high representativeness and a factor loading higher than 0.40 (absolute value) indicating significant contribution of the item. Internal consistency of the questionnaire and each of its a priori dimensions and sub-dimensions was measured by the Cronbach’s alpha coefficient (if alpha > 0.70, consistency was considered as satisfactory). As a result of the analysis, the questionnaire was trimmed and a new construct of dimensions and sub-dimensions was envisaged.
- For the confirmatory analysis, the Structural Equation Model (SEM) was used to confirm the construct assumption of the questionnaire into dimensions and sub-dimensions, and the adjustment of the model to the data [43]. The SEM consisted in an external model representing the relationships between the latent (the dimensions) and manifest variables (their items), and an internal model representing the relationships between the latent variables. The Partial Least Square (PLS) approach was used to estimate both the external and internal models. This approach allows determining latent variables without any assumption on distribution. The SEM model allowed calculating scores for each latent variable and taking into account the latent variables associated items and the causal ratio with the other latent variables [44, 45].
  Dillon-Goldstein’s rho coefficient was calculated for each sub-dimension to confirm internal consistency as established by the SEM (threshold at 0.70). External quality of the models was measured by the Goodness of Fit (GoF) criterion. The predictions on the external model gave standardised external weights for quantification of the impact of items on their sub-dimension (percentage of the item effect on the sub-dimension). Finally, the calculation of structural coefficients allowed to make predictions on the internal model and to rank the sub-dimensions on the basis of their impact on OC.
- A test-retest was performed to assess reproducibility. OC questionnaire was collected twice by the same group of health care professionals at two times separated by three weeks. For each item a percentage of agreement and an Intra-Class Correlation Coefficient (ICC) (of the 1.1 type) were calculated, reproducibility was good when the ICC value was higher than 0.70 [46].

## Results

### OC questionnaire construct

The literature review identified 41 OC measuring instruments. The expert group selection process excluded all but 14, out of which a base of 906 items and 100 themes was created. Were extracted and removed from this database the items that were less relevant to a French context, mainly a few items clearly oriented to productivity and on fierce competition. Healthcare professionals are not confronted with direct competition and productivity requirements in the French healthcare system. It is at least partly explained by the modalities of hospital professional management, based on public sector rules and by patient management means, organized at ward level. Items that could not be found in selected questionnaires were introduced as they were considered key points in sociology of work. They were items on clinical decision making and others related to discriminatory practices (according to specific cultural or religious orientation or to gender).

All the instruments that were selected contributed to the questionnaire construct (Table 2). Eight different OC dimensions were identified. Under those dimensions, 26 sub-dimensions were identified and 131 items were spelled out. Three validated scales were retained in their entirety: professional life satisfaction scale (5 items) [47, 48], intention to stay scale (6 items) [49], and professional burn out scale (6 items) [50]. They all contain 17 items.

**Table 2:**
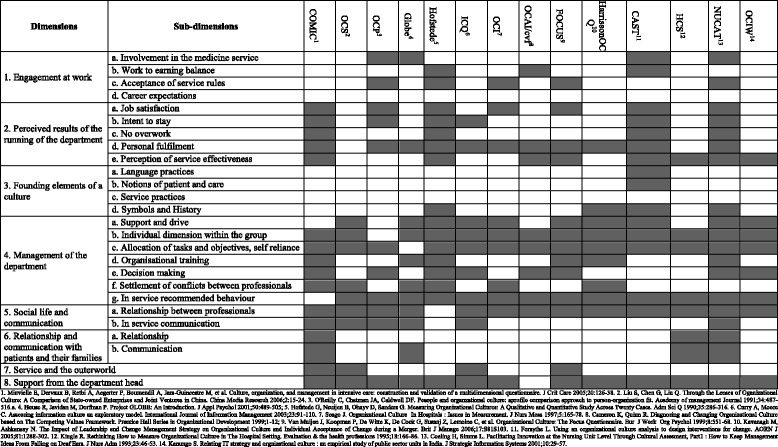
Dimensions and Sub-dimensions of the new classification and approaches taken by the various instruments under analysis

Face validation led to some changes in item wording. Sub-dimensions “Conflict management procedures” and “Frequency of conflicts between professionals” were both treated separately for doctors and for paramedical professionals. The same was done for the sub-dimension “Quality of relationship between professionals” with creation of two new sub-dimensions: “Relations between paramedical professionals” and “Relations with and between doctors”.

### Data collection

A total of 36 medicine wards from 33 hospitals in the five French regions participated; there were 7 university hospital wards, 9 large hospital wards, 14 small hospital wards and 6 private hospital wards. The OC questionnaire was distributed to a total of 1081 professionals working in those wards (176 doctors, 440 nurses, and 465 orderlies).

A total number of 859 questionnaires were returned (79 % return rate, 77 % for doctors, 82 % for nurses, and 78 % for orderlies). There was no noticeable difference in participation rate per region. Table 3 shows the socio-demographic characteristics of the study sample. The vast majority of orderlies and nurses were women (respectively 93.8 and 92.5 %) with average ages under 40 years (respectively 39 – Standard Deviation SD: 9 and 37 – SD: 9). They both were most frequently working at day time only (respectively 81.5 and 63.9 %) with permanent status (71.6 % for both); the majority (57.8 %) of medical doctors were men with an average age of 42 years (SD: 11). In term of seniority, nurses had an average of 6 years of tenure (SD: 6), while orderlies and medical doctors had averages of 13 years (SD: 9.7) and 15 years (SD: 10.6), respectively.

**Table 3:** Socio-demographic characteristics of the sample population of professionals

	Orderlies		Nurses		Medical doctors	
	*N* =362		*N* =361		*N* =136	
	Yes	%	Yes	%	Yes	%
Women	334	93.8	332	92.5	57	42.2
Age						
Average (standard deviation)	39 (9)	-	37 (9)	-	42 (11)	-
Median (min-max)	40 (20–59)	-	35 (21–61)	-	42 (23–65)	-
From 20 to 29	68	19.8	106	30.6	26	19.3
From 30 to 39	103	30.0	118	34.0	32	23.7
From 40 to 49	110	32.1	77	22.2	38	28.1
> = 50 years of age	62	18.1	46	13.3	39	28.9
Time slot						
Daytime only	291	81.5	228	63.9	-	-
Nights only	42	11.8	59	16.5	-	-
Day and night	24	6.7	70	19.6	-	-
Status						
Permanent	254	71.6	254	71.6	-	-
Locum tenens	28	9.8	28	9.8	-	-
Interim staff	5	1.9	5	1.9	-	-
Seniority in the service						
Average (standard deviation)	12.8 (9.7)	-	5.5 (6)		14.6 (10.6)	-
Median (min-max)	10 (1–40)	-	4 (0–31)		13 (0–36)	-
From 1 to 10	166	50.6	279	85.1	50	40.7
From 11 to 20	82	25.0	36	11.0	32	26.0
From 21 to 30	61	18.6	12	3.6	32	26.0
From 31 to 40	19	5.8	1	0.3	9	7.3
Continuous education (during professional activity)						
Less than one year	203	58.2	237	66.8	109	80.7
From 1 to 5 years	110	31.5	87	24.5	10	7.4
From 5 to 10 years	11	3.2	8	2.3	0	0.0
Over to 10 years	2	0.6	0	0.0	0	0.0
No continuous education	23	6.6	23	6.5	16	11.9

Most represented medical specialities were cardiology (19.8 %), geriatrics (13.2 %), internal medicine (13.2 %), and also though to a lesser extent, nephrology (9.9 %) and hepato-gastro-enterology (9.9 %). Doctors’ positions were ventilated as follows: service head doctors (13.2 %), university professors (PU) or university professors/ hospital clinicians (PU-PH) (36.4 %), attendants (5.4 %), consultants (3.1 %), interns (17.1 %), clinicians on salary (10.9 %), private practitioners (14.0 %)

### Exploratory analysis

Table 4 presents the main results of the exploratory analysis.

**Table 4:** Metrological properties of the first version of the organizational culture questionnaire

Heading	Nb of items	Response rates	Saturation	Correlations		Overall PCA (31 factors)	Cronbach Alpha values	
				<0.20	>0.60			
1. Engagement at work								
a. Engagement with hospital department	6	(97.2–99.8)	Ceiling effect (3)	(2)		F3(1) **F12(3)** F24(1) F31(1)	0.668	↘ (1)
b. Work to earning balance	4	(99.0–99.2)	Ceiling effect (1)	(3)		**F22(3)** F31(1)	0.393	↘ (1)
c. Acceptance of department standards	4	(98.4–99.2)	Ceiling effect (1)	(4)		**F10(2)** F31(1)	0.575	↘ (1)
d. Professional aims	5	(99.1–99.8)	Ceiling effect (5)			**F2(5)**	0.712	
2. Perceived results of the running of the department								
a. Satisfaction at work	5	(99.2–99.5)			2	**F11(5)**	0.840	
b. Likelihood of remaining on staff	6	(98.5–99.1)	Ceiling effect (4)		5	**F3(6)**	0.911	↘ (1)
c. Workload	8	(98.3–99.1)	Ceiling effect (1)	(1)		**F6(5)** F9(1) **F29(2)**	0.777	↘ (1)
d. Work exhaustion	5	(97.6–99.0)		(1)		F5(1) F6(1) F9(2) F11(1)	0.697	↘ (1)
e**.** Perceived efficiency of the department	4	(98.0–98.6)				**F17(4)**	0.747	
3. Founding elements of a culture								
a. Language practices	4	(98.5–99.3)	Ceiling effect (1)	(1)		F4(1) F19(3)	0.414	↘ (1)
b(a). Notions of patient and care	3	(97.7–98.4)		(2)		F4(1) F13(1) F21(1)	0.473	↘ (1)
b(b). Service practices	3	(98.0–98,6)	Ceiling effect (1)	(2)		F7(7) F28(1)	0.447	↘ (1)
c. Symbols and History	3	(97.4–97.7)				F14(3)	0.723	
4. Service Management of the department								
a. Support and drive	2	(97.8–98.0)			1	**F1(2)**	0.916	
b. Taking account of the individual in the group	7	(96.9–98.3)	Ceiling effect (1)	(6)		**F1(2) F23(4)**	0.633	↘ (3)
c. Allocation of tasks and goals	4	(97.3–98.4)	Ceiling effect (1)	(1)		**F21(3)**	0.630	↘ (1)
d. Organisational training	2	(97.3–97.9)		(2)		F1(1)	0.350	
e. Decision making	2	(97.3–97.6)				**F8(1)**	0.646	
f. Management of conflicts between professionals	9	(96.7–97.7)		(1)		**F27(4) F30(5)**	0.807	↘ (1)
g. Type of behaviour encouraged in the department	5	(96.6–97.9)	Ceiling effect (2)	(3)		F24(1) **F18(4)**	0.526	↘ (1)
5. Relation and communication in the department								
a. Relationship between professionals	13	(94.0–97.4)		(4)	1	F4(2) **F8(5) F16(3)** F20(2) F27(1)	0.834	
b. Diffusion of information	6	(96.6–97.4)		(1)	1	**F15(3) F28(2)**	0.696	↘ (1)
6. Relationship and Communication with patients and their families								
a. Relationship	3	(97.3–97.9)	Ceiling effect (1)	(2)		**F13(3)**	0.491	↘ (1)
b. Communication	2	(97.3–97.7)				**F13(2)**	0.549	
**7. Service and the outerworld**	3	(96.7–97.7)		(2)		F26(2)	0.470	
8. Support by the service head doctor	13	(94.8–95.1)		(2)	6	**F5(9)** F25(4)	0.872	

NB:

▪ Nb of items: Number of items in the sub-dimension before item removal

▪ Response rates: Range of response rates over the sub-dimension items

▪ Saturation: Number of items, in parentheses, producing a ceiling effect (more than 30 % of level 5 responses)

▪ <20 Correlation: Number of items having a lower than 0.20 correlation with at least half of the other items in the sub-dimension

▪ >60 Correlation: Number of correlations higher than 0.60 between two items

▪ Overall PCA: List of factors and corresponding number of items in parentheses on which items had some impact, as demonstrated by the PCA analysis and after the *oblimin* rotation (contribution >0.40); in bold, factors that were maintained in the validated version

Cronbach Alpha values: values indicated per sub-dimension and indication of the number of items necessary to bring the sub-dimension value down

Professionals completed the questionnaire in 15 min in mean. The item response rate varied between 94 and 99 % and in more than 90 % of the cases all the items were completed. 22 items had a ceiling effect. Several items had a low correlation with all the others in the same sub-dimension and with most in the other sub-dimensions. Two items were redundant.

The PCA pinpointed 31 factors. The questionnaire’s unidimensional character was confirmed. In some sub-dimensions, such as 2a- “Satisfaction at work” and 2b- “Likelihood of remaining on staff”, all the items appeared on the same PCA factor after rotation, while in others, such as 3b(a)- “Notion of patient and care”, the items were all on different factors. However, in most cases, a dominant factor appeared and the items that were not on that factor were either irrelevant or in the wrong place. 38 items were multidimensional and 22 items did not appear on their sub-dimension main factor.

The Cronbach's alpha was of 0.95 for the questionnaire and ranged from 0.47 to 0.89 for dimensions and 0.35 to 0.92 for sub-dimensions. Internal consistency was considered as satisfactory in ten sub-dimensions. In 15 other sub-dimensions, removing one or several items increased the alpha coefficient.

On the basis of the results, a new structure of 21 sub-dimensions and 6 dimensions was devised. Table 5 presents the psychometric properties of the final version of the questionnaire. Cronbach’s alpha of this modified version of the questionnaire ranged from 0.54 to 0.91 for sub-dimensions. Internal consistency was considered as satisfactory in ten sub-dimensions. The Cronbach’s coefficients remaining under 0.70 corresponded to 8 sub-dimensions of which 5 contained only two or three items. One sub-dimension had a Cronbach’s alpha lower than 0.6. Two dimensions and nine sub-dimensions were deleted. Five sub-dimensions were split and two have been merged. 52 items have been deleted and 13 were rephrased. For example, all items expressed in a negative way were rephrased to items expressed with a positive way. Finally four items were added to maintain the sub-dimension « 4D – Organisational learning », that have been excluded at the confirmatory analysis step.

**Table 5:** Metrological properties of the validated version of the organizational culture questionnaire (final version)

	Exploratory analysis					Confirmatory analysis		
Heading	Nb of items	Nb of rephrased items	Overall PCA (20 factors)	Cronbach Alpha values		Dillon-Goldstein’s Rho coefficient	Structural coefficient	Sub-dimension Ranking (impact on OC)
1. Engagement at work								
a. Engagement with hospital department	5	2	F14(3) F18(2)	0.675		0.796	0.083	8
c. Acceptance of department standards	2	0	F7(2)	0.695		0.867	0.020	20
d. Professional aims	5	0	F3(5)	0.712		0.825	0.026	19
2. Perceived results of the running of the department								
a. Satisfaction at work	4	0	F5(4)	0.785		0.863	0.113	6
b. Likelihood of remaining on staff	2	0	F18(2)	0.826		0.919	0.114	4
c(1). Workload	2	0	F19(2)	0.534		0.813	0.045	17
c(2). Work exhaustion	5	0	F2(5) F19(1)	0.789		0.858	0.057	14
e. Perceived efficiency of the department	4	1	F11(4)	0.747		0.845	0.067	11
4. Management of the department								
b(1). Taking account of the individual in the group	4	1	F15(4)	0.795		0.870	0.125	3
b(2). Discrimatory practices	2	2	F13(2)	0,525		0.823	0.032	18
c. Allocation of tasks and goals	3	1	F16(3)	0.707		0.838	0.069	10
d. Organisational learning	4	4						
f(1). Frequency of conflicts between professionals	4	0	F6(4) F1(2)	0.766		0.853	0.114	4-bis
f(2). Conflict management	5	1	F20(5) F6(1)	0.743	↘ (1)	0.835	0.111	7
g. Type of behaviour encouraged in the department	4	0	F10(4) F15(1)	0.635	↘ (1)	0.789	0.049	16
5. Relation and communication in the department								
a(1). Relations between paramedical professionals	3	1	F8(3) F6(1)	0.682	↘ (1)	0.827	0.065	12
a(2). Relations with and between doctors	6	2	F1(6) F13(1)	0.831		0.881	0.159	2
b(1). Coordination within the department	2	0	F17	0.672		0.863	0.075	9
b(2). Diffusion of information	3	0	F9	0.743		0.855	0.058	13
6. Relationship with patients and their families	5	2	F12	0.607		0.757	0.057	14-bis
8. Support from the department head	9	0	F4	0.908		0.928	0.223	1

NB:

▪ Nb of items: Number of items in the sub-dimension of the validated version

▪ Nb of rephrased items : Number of rephrased items between initial and final (validated) version

▪ Overall PCA: List of factors and corresponding number of items in parentheses on which items had some impact, as demonstrated by the PCA analysis and after the *oblimin* rotation (contribution >0.40). The sum of number of items in parentheses may be over to the number of items of the sub-dimension if an item contributes to several factors

▪ Cronbach Alpha values: values indicated per sub-dimension and indication of the number of items necessary to bring the sub-dimension value down

▪ Structural coefficient : the figures indicate the ranking (from the highest to the most little) of the dimensions through MES type PLS

▪ Dillon-Goldstein’s Rho coefficient: values indicated per sub-dimension, value >0,70 is considered as satisfactory

### Confirmatory analysis

A SEM was applied to the 79 items regrouped into 20 sub-dimensions and 6 dimensions (without “Organizational learning” sub-dimension). The Dillon-Goldstein’s rho coefficient ranged from 0.76 and 0.93 (Table 5). The external model quality was high (GoF index equal to 0.99).

The standardized external weights per item varied from 0.08 to 0.86. There was little impact difference between the items of each sub-dimension except in 1c- “Acceptance of department standards”, 1d- “Professional aims”, 2c(2)- “Work exhaustion”, 4b(2)- “Discriminatory practices” and 4 g- “Type of behaviour encouraged in the department”, where one or several items had considerably less impact than the others. The internal model structural coefficients allowed ranking the sub-dimensions as a function of their impact on the OC concept (Table 5). The sub-dimensions which had the greatest impact on OC concept were: “Support from the department head” (Structural Coefficient– SC: 0.223), “Relations with and between doctors” (SC: 0.159) and “taking account of the individual in the group” (SC: 0.125). Dimensions having the smallest impact on OC concept were: “Acceptance of department standards” (SC: 0.020), “Professional aims” (SC: 0.06) and “Discrimatory practice” (SC: 0.032).

**Fig. 1:**
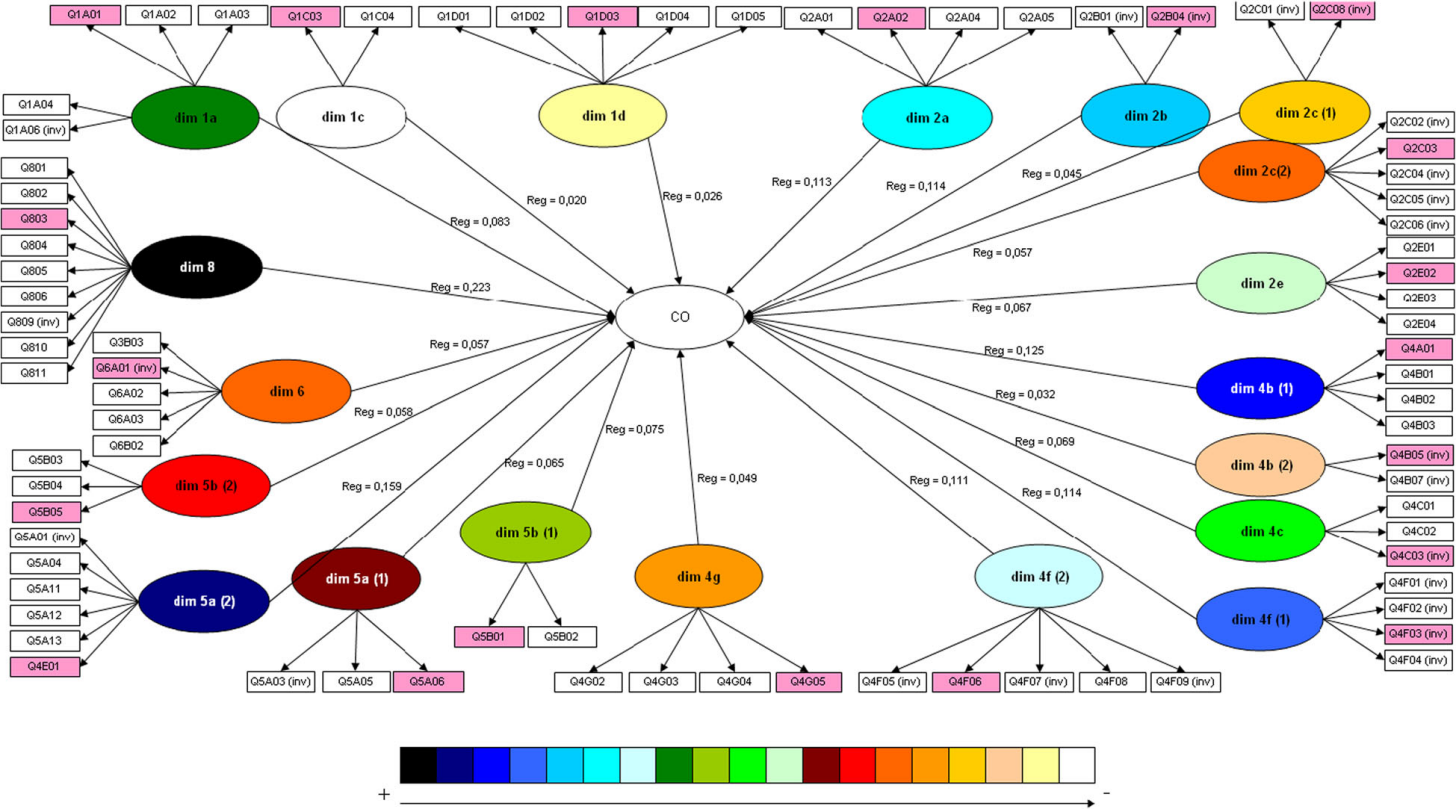
Confirmatory factor analysis of the hypothesized structure of the CO questionnaire: impact of the manifest variables on the latent variables and of the latent variables on the Organizational Culture. Rectangles represent items; circles represent the dimensions (dim) and the organizational culture, or latent variables. ‘Reg’ is the structural coefficient of each dimension on the organizational culture. The color variation (from white to dark) shows which dimensions have the most impact on the organizational culture

Figure 1 corresponds to the SEM’s graphical representation linking the dimensions and the items of the questionnaire. The dimensions are latent variables represented by circles and the items are manifest variables represented by rectangle. For every sub-dimension, the standardized external weight by every item was calculated. The items having the greatest impacts on its dimension are presented in pink. All the latent variables converge on a central concept, the organizational culture. The structural coefficient (Reg) allowed ranking the sub-dimensions as a function of their impact on the organizational culture concept; the biggest were the one that had the greatest impact (colour shading).

### Reproducibility analysis

The questionnaire which was administered at that stage included 83 items and 21 sub-dimensions. The participating department was a medicine ward with 19 professionals, 13 of whom responded twice. For 70 % of the items, reproducibility was average (25 items) to good (33 items). For three items, the ICC could not be calculated. Six sub-dimensions had more than half of their items with an ICC lower than 0.50, two of them were related to the perception of results of the running of the department (2b- “Likelihood of remaining on staff”, 2c(1)- “Workload”), three of them were department management components (4c- “Allocation of tasks and goals”, 4d- “Organisational training”, 4f(1)- “Frequency of conflicts between professionals”), and the sixth one was 5b(2)- “diffusion of information”.

## Discussion

The comprehensive construct, the one-dimensional character and the reliability of COMEt questionnaire suggest a good validity of the instrument for interventional research.

The high overall response rate of 80 % indicates its applicability and acceptability in interventional research conditions. The item selection process allowed the deletion of 52 items, resulting in a final version with 82 items. The challenge of questionnaire validation is to keep the questionnaire as short as possible while still including enough items to adequately measure the variables of interest. The validation results show that this challenge was successfully met.

Internal consistency was globally satisfactory. The retention of three entire scales (professional life satisfaction scale, intention to stay scale and professional burn out scale) has strengthened this psychometric property. Only eight sub-dimensions had Cronbach’s coefficients under 0.70, with five of them containing only two or three items; that is a factor of Cronbach’s coefficient decreasing. Furthermore, we expect those coefficient would increase after taking into account the final rewordings of the final version of the questionnaire (COMEt final version translated in English presented in Additional file 1).

Dillon–Goldstein’s rho coefficients higher than 0.70 for the sub-dimensions, indicate a high homogeneity of the instrument and more than 70 % of the items have a moderate to good reproducibility index.

COMEt construct followed a strict pattern covering a maximum number of OC dimensions and sub-dimensions. As a result of a comprehensive search of existing tools in literature, our instrument is the only one to cover all the OC components treated in the 14 retrieved OC questionnaires. Those 14 tools include most of the instruments retained by T Scott et al. [51] in their descriptive review of available instruments and they also appear in T Jung et al’s inventory of instruments for exploring OC [37].

With such a large number of OC dimensions, our OC questionnaire, provides, in addition to be an intervention assessment tool for research purposes, an operational tool for precise and comprehensive managerial and organizational diagnosis at ward level, thus allowing the identification of priorities of change to improve attitudes, effectiveness and performance. COMEt results have been indeed presented to staff and managers of several participating wards. The wards’ leaders mainly thought COMEt results were concordant with their perception of team climate in their ward and that they help them to adjust their managerial strategies.

M Sibe et al. published an analysis of data collected for COMEt validation that reinforces its internal and external validity [52]. M Sibe et al. showed the discriminative capacity of COMEt instrument. The analysis of responses obtained for COMEt validation, allowed to distinguished two groups of wards: the wards with high development of all COMEt dimensions (9 wards on the 36 included, called “optimistic” wards) and the wards with low development of the COMEt dimensions (13 wards on the 36 included, called “pessimistic” wards). There was also a third group of 12 wards with intermediate results. One of the critical functions of a group’s culture is to establish a distinctive identity and thereby provide a means by which members of the group can differentiate themselves from other groups. COMEt showed its discriminative capacities, in accordance with this property of OC. Moreover, these results give arguments for an external validity of Comet. They are in favour of the strength of the existence of an organizational context that can induce either a positive or a negative effect both on management practices and attitudes; that is in coherence with magnet hospital theory change for management and the explanation of the link between OC and performance.

Questions may rise about the organizational issues assessed by COMEt. The first one concerns whether it really captures OC or rather organizational climate. COMEt investigates into layers 3 (artefacts) and 2 (values) of culture as defined by Schein. It is probably a poorly adapted tool for layer 1 assessment, that is the “assumption” level; actually that limitation appears in all the questionnaires, and it is thought to be a consequence of the questionnaire method [29]. In their recent comparative literature review on organizational climate and culture, B Schneider et al. acknowledge that “what most quantitative measures of culture capture are the espoused values and or behavioural norms in organizations and not the full richness of the construct, including myths, stories, and socialization tactics” [15]. Indeed, culture attempts to address deeper values and assumptions rather than the surface perceptions that are the focus of climate studies assessing specific process and content dimensions of behaviour. OC also emphasises that which is shared by group members rather than the diversity of individual perceptions that can make up climate [51]. However, the theoretical distinction between culture and climate is not always upheld in practice. Schein has more recently characterized climate as providing the behavioural evidence for the culture of a setting, such that those behaviours form the bases for employees’ conclusions about the values and beliefs that characterize their organization. Culture and climate are now considered as two crucial, complementary and interrelated building blocks for organizational description and analysis [53]. Our tool collects the shared participants’ views of the environment in which they work. In its main dimensions and sub-dimensions, it measures the shared values and artefacts of a department, or the “visible” or “expressive” part of the culture; it also captures specific process and content dimensions of behaviour, retrieved in some items of the dimensions “Management of the Department” or “Relation and communication in the department”, characterizing “climate”. This integration of climate and culture assessment has useful implications for practice and organizational changes and for research on the link between organization and performance.

A second question concerns the specificities of COMEt questionnaire in comparison with team work questionnaires. A recent review retrieved survey instruments used to assess dimensions of teamwork [54]. All but one retrieved teamwork dimensions assessed by surveys are found in COMEt questionnaire. However, COMEt questionnaire contains supplementary dimensions and sub-dimensions relative to professionals’ attitudes and management practices (“Relations with the patient and his/her family”, “Type of behaviour encouraged in the department”, “Allocation of tasks and goals”, “Engagement at work”, “Discriminatory practices” and “Likelihood of remaining on staff”). There is clearly overlapping between COMEt OC assessment and team work assessment. However, COMEt specifically collects professional attitudes to work and managerial practices.

COMEt was validated within medicine wards for the TheOReM© project’s purposes. This specificity is a current limitation to the instrument. Validation of this questionnaire in other type of wards (surgery, other medicine specialities) is ongoing in France. The validation of the questionnaire in other health systems and other language is also needed. The trans language validation of the Hospital Survey on Patient Safety Culture questionnaire showed that a core set of dimensions consistently assessed as valid can be defined and measured in all countries and that some other should be adapted to each country according to local ways of being, thinking, behaving and communicating [55]. The extent to which the COMEt questionnaire is specific to the French context can not be known without such translation validation. COMEt development included specificities of French health care system that would probably lead to exclude some items from a COMEt international common core set. From our comprehensive database of OC items and themes, were extracted and removed the items which were less relevant to a French context (mainly few items clearly oriented to productivity, items on fierce competition). Face validation led to some changes in item wording that are probably a direct consequence of French operational specificities. As example, contrary to what could be found in other instruments, conflict management procedures or quality of relationship between professionals were treated separately for medical doctors and paramedical professionals. In a transvalidation process across language and health care systems, attention should be put on these items. Moreover, items relative to productivity or competition should perhaps be added.

We did not study the sensitivity to change of the questionnaire. We aim to study this property as part of an ongoing research studying the impact of tools aiming to improve development of patient centred care at hospital using organizational and managerial changes and to analyze the individual and organizational determinants of their effectiveness. It focuses on 30 acute care hospitals that have the choice of the combination of tools and that are followed during two years with combined quantitative and qualitative analyses. That project uses the COMEt questionnaire for ward organizational context assessment. The comparison of the COMEt questionnaire dimensions scores before and after implementation of the tools and between departments implementing different tools will allow the analysis of COMEt questionnaire sensitivity to change.

The validation strategy was to proceed to exploratory and confirmatory analyses using the PLS (Partial Least Square) approach [42]. Very few studies refer to a subsequent confirmatory analysis. The only example we could find was an Australian publication [56] which relates the use of SEM in the confirmatory phase, to validate an organizational culture measuring instrument. There are two different SEM types which permit confirmatory analysis but they differ in principle, purpose, rules and applications [57]. Contrary to the Australian who adopted the LISREL (LInear Structural RELation) approach, we opted for the PLS approach in our study because it enabled us to predict individual dimension scores. It is the first step of the confirmatory analysis which will be completed by a LISREL approach trough a planned cross validation study including another sample from different wards specialties. This cross validation also will be able to test the performance of the reformulated items, particularly those of the sub-dimension “organizational learning”.

The SEM confirmatory analysis indeed gives distinctive and relevant information: quantifying items impact on its sub-dimension and ranking sub-dimensions impact on the organizational culture concept. The dimensions which had the strongest impact were “Support from the department head” followed by dimensions related to professional interaction and those related to well being within the group. Dimensions related to work, tasks and performances (workload, discriminatory practices, kind of behaviour encouraged in the department) had a lower impact weight on the organizational culture concept. The validation study of the French version of the Hospital Survey on Patient Safety Culture showed consistently that the three dimensions having the strongest impact on safety culture were: ‘Overall perceptions of safety’, ‘Hospital management support for patient safety’ and ‘Teamwork across hospital units’ [57].

From those findings one could draw the conclusion of a very emotional work perception culture, value is given to wellbeing within the group and to friendly climate. The dimension hierarchy as established by the analysis provides substantial information in favour of the hypotheses that changes into organizational culture are to be introduced by the management staff. In that type of medicine department, action must undoubtedly be focused on the group, on its internal harmony and on the integration of professionals within the group.

## Conclusions

The COMEt questionnaire is a validated multidimensional OC questionnaire made of 6 dimensions, 21 sub-dimensions and 83 items. It is the first multidimensional OC questionnaire, specific to healthcare context, for a unit level assessment showing robust validity and reliability. This tool is suited for research purposes, especially for assessing organizational context in research analysing effectiveness of hospital quality improvement strategies [58]. Our tool is also suited for an overall assessment of ward culture for team-training activities and could be a powerful trigger to improve management and clinical performance. Its psychometric properties in other health systems need to be tested.

## Additional file

Additional file 1: COMEt questionnaire in English. Description of data: Presentation of the final version of the COMEt questionnaire, translated from French to English. (DOC 120 kb)

## Abbreviations

CCECQA: Comité de Coordination de l’Evaluation Clinique et de la Qualité en Aquitaine
COMEt: Contexte Organisationnel et Managérial en Etablissement de santé
GoF: Goodness of Fitness
HAS: Haute Autorité de Santé
ICC: Intra-Class Coefficient
LISREL: LInear Structural RELations
OC: Organizational Culture
PCA: Principal Component Analysis
PLS: Partial Least Square
SEM: Structural Equation Model
SC TheOReM: Organizational theOries, Recommendations and Management
