# Supplementary material for: Design and validation of a questionnaire to assess organizational culture in French hospital wards
Source: BMC Health Serv Res. 2016 Sep 17;16:491. doi: 10.1186/s12913-016-1736-4 (PMC5027118; doi:10.1186/s12913-016-1736-4)
Supplement: Additional file 1: — COMEt questionnaire in English. Description of data: Presentation of the final version of the COMEt questionnaire, translated from French to English. (DOC 120 kb) [file 12913_2016_1736_MOESM1_ESM.doc]

**COMEt Questionnaire in English**

| **1 - Engagement at work** |
| --- |
| **Engagement with hospital department** |
| I am very emotionally attached to this department |
| I work in this department through personal choice |
| My work has absolute priority |
| Problems in the department are my problems |
| I am committed to this department |
| **Acceptance of department standards** |
| I never disagree with my superior |
| I am happy to respect the rules in force in my work |
| **Professional aims** |
| It is important for me to ensure the well-being of the patient and his/her family |
| It is important for me that the department should be well managed and organised |
| It is important for me that there are good working relationships with my colleagues |
| It is important for me that I am efficient in my job and that patients should be treated as quickly as possible |
| It is important for me to defend the benefits of my profession |
| **2. Perceived results of the running of the department** |
| **Satisfaction at work** |
| In most respects, my professional life is close to my ideal |
| My working conditions are satisfactory |
| So far, I have achieved the most important things (for me) that I wanted to achieve in my professional life |
| If I could have my professional life over again, there is almost nothing I would change |
| **Likelihood of remaining on staff** |
| I have no plans to leave this department or this hospital |
| There is nothing forcing me to stay in this department |
| **Workload** |
| At work, I am not often overwhelmed due to lack of time |
| I do not have too many patients in my care |
| **Work exhaustion** |
| When I get up in the morning, I do not think about work problems |
| When I go home, I find it easy to relax and switch off from work |
| Those close to me do not think that I make too many sacrifices for my work |
| I am not anxious about my work, I do not worry about it when I go to bed |
| If I put off until tomorrow something that I should have done today, this will not disturb my sleep |
| **Perceived efficiency of the department** |
| We obtain good results for the patients we treat |
| Our department responds correctly to the needs of the patients' family members |
| Professionals in the department (orderlies, doctors) are among the best |
| We manage to keep the best professionals (orderlies, doctors) in our department |
| **3. Management of the department** |
| **Taking account of the individual in the group** |
| Professionals feel that their work is appreciated by their supervisor |
| Professionals' opinions and ideas are taken into account when deciding on patient care |
| Professionals' opinions and ideas are taken into account when taking strategic decisions in the department |
| Professionals are able to question a decision by a superior |
| **Discriminatory practices** |
| The allocation of roles and responsibilities in the department never depends on whether one is a man or a woman |
| The allocation of roles and responsibilities in the department never depends on cultural characteristics |
| **Allocation of tasks and goals** |
| Professionals' activities and tasks are clearly defined and allocated |
| The goals of each professional are identified and assessed |
| In the department, rules about the allocation of tasks are always respected |
| **Organizational learning** |
| Professionals implement actions to improve health quality and safety |
| When improvement actions are implemented, their effectiveness is assessed |
| Dysfonctions within the ward are not enough analysed |
| Professionnals learn quickly when they are confronted with new problems |
| **Frequency of conflicts between professionals** |
| In the department, there are few disagreements between doctors |
| In the department, there are few disagreements between paramedical professionals |
| In the department, there are few disagreements between doctors and paramedical staff |
| When there are disagreements between professionals, these differences of opinion rarely cause arguments |
| **Conflict management** |
| If there are disagreements between professionals, the problem is ignored |
| If there are disagreements between professionals, all points of view are considered in order to reach an agreement |
| If there are disagreements between professionals, it is always the same person who decides |
| If there are disagreements between professionals, the professionals try to find a solution |
| If there are disagreements between professionals, each professional tries to ensure that they come off best |
| **Type of behaviour encouraged in the department** |
| Professionals are encouraged to respect protocols and recommendations in force meticulously |
| Professionals are encouraged to take initiatives |
| Professionals are encouraged to be efficient and productive |
| Professionals are encouraged to work in teams |
| **4. Relations and communication in the department** |
| **Relations between paramedical professionals** |
| Paramedical professionals are not openly competitive |
| Professionals on the same professional level help one another |
| Professionals trust one another |
| **Relations with and between doctors** |
| Medical doctors are not openly competitive |
| Professionals (doctors and paramedicals) form a solid team |
| Doctors respect the paramedical professionals |
| Doctors are willing to listen to advice from paramedical professionals |
| Paramedical professionals have great respect for the doctors |
| Medical decisions about patient care are taken collectively |
| **Coordination within the department** |
| In the department, meetings are the opportunity for really getting together, for discussion and exchange |
| When operational changes are decided on, they are explained clearly |
| **Diffusion of information** |
| Oral information sent to professionals is not ambiguous |
| Written information sent to professionals is not ambiguous |
| Information circulates freely between professionals |
| **5. Relations with the patient and his/her family** |
| The importance paid to the relationship with the patient is shared by the entire department |
| When professionals are with a patient, they rarely speak to each other as if the patient didn't exist |
| Professionals are attentive to the needs of patients and their families |
| Patients' cultural differences can be taken into consideration when they are cared for |
| Professionals caring for patients spend time educating the patient and his/her family |
| **6. Support from the department head** |
| The department head has a vision for the future needs of the department |
| The department head stimulates and mobilises the professionals in their daily routines |
| The department head is able to spend time helping the others |
| The department head is able to bring professionals round to his/her point of view |
| The department head is fair, honest and inspires confidence |
| The department head takes decisions that are decisive, quick and coherent |
| The department head does not act in his/her personal interest |
| The department head attaches importance to the well-being of the professionals in the department |
| The department head establishes friendly relations with the professionals in the department |
